# Supplementary material for: Bacterial Toxins from Staphylococcus aureus and Bordetella bronchiseptica Predispose the Horse’s Respiratory Tract to Equine Herpesvirus Type 1 Infection
Source: Viruses. 2022 Jan 14;14(1):149. doi: 10.3390/v14010149 (PMC8778808; doi:10.3390/v14010149)
Supplement: Supplementary file 1 [file viruses-14-00149-s001.zip › viruses-1536325-supplementary.pdf]

Supplementary information

Bacterial toxins from *Staphylococcus aureus* and *Bordetella bronchiseptica* predispose the horse's respiratory tract to equine herpesvirus type 1 infection

Van Crombrugge, E., Vanbeylen, E., Van Cleemput, J., Van den Broeck, W., Laval, K. and Nauwynck, H.J.

Supplementary figures

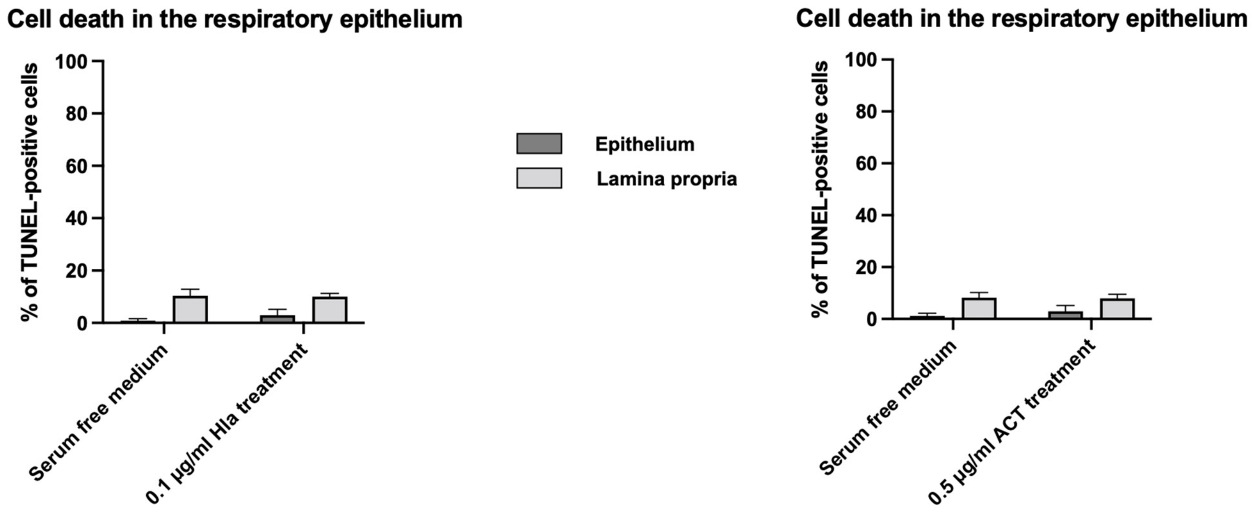

Figure S1. Cell viability in respiratory mucosal explants

The viability of the respiratory mucosa (epithelium and lamina propria) after treatment with the Hla (left) and ACT (right) toxins was assessed by means of TUNEL stainings. Data are represented as mean values + SD of triplicate independent experiments. No substantial increase in the amount of TUNEL-positive cells was observed in the respiratory mucosa (i.e. epithelium and lamina propria) after treatment with 0.1 µg/ml Hla toxin or 0.5 µg/ml ACT toxin.
